# The Role of Machine Learning in Addressing Antibiotic Resistance: A New Era in Infectious Disease Control

**DOI:** 10.1002/mbo3.70160

**Published:** 2025-11-24

**Authors:** Akmal Zubair, Mohd Fazil, Muhammad Jawad, Safa Wdidi

**Affiliations:** ^1^ Department of Biotechnology Quaid‐i‐Azam University Islamabad Pakistan; ^2^ College of Computer and Information Sciences Imam Mohammad Ibn Saud Islamic University (IMSIU) Riyadh Saudi Arabia; ^3^ Faculty of Medical Sciences Laboratory, Oncology Research Center University of Shendi Shendi Sudan

**Keywords:** antimicrobial resistance, artificial intelligence, bacteria, drugs, models

## Abstract

Antimicrobial resistance (AMR) refers to the ability of microorganisms, such as bacteria and viruses, to resist antimicrobial medications. Current strategies for addressing this escalating issue are often labor‐intensive and costly. However, advancements in artificial intelligence (AI) are enabling the rapid evaluation of extensive chemical libraries and the prediction of novel antimicrobial compounds. AI holds significant promise for medical research in treating multidrug‐resistant infections by enhancing the development of new medications through the analysis of antibiotic usage, disease prevalence, and resistance patterns. The use of AI has the potential to greatly benefit research by accelerating the discovery of new antibiotics that effectively combat antibiotic‐resistant microbes. Predicting trends in antibiotic resistance through the examination of large data sets by AI systems may pave the way for the creation of preventative medicines. The speed and accuracy with which AI can evaluate data are revolutionizing how scientists develop new medicines, assess potential health concerns, and find ways to prevent illness. AMR is a growing concern, and AI is playing an increasingly crucial role in this battle. Medical research utilizing AI has tremendous potential in the ongoing fight against antibiotic resistance. This review examines how AI aids in AMR diagnosis, small molecule drug development, and the detection of AMR symptoms. Further research into AMR detection and the creation of novel medications are two areas that could prove valuable in treating antimicrobial resistance.

## Graphical abstract

Artificial intelligence accelerates the discovery of new antibiotics by rapidly analyzing vast chemical libraries to combat antimicrobial resistance effectively. AI systems predict antibiotic resistance trends, helping researchers design targeted therapies, and prevent the spread of multidrug‐resistant infections.

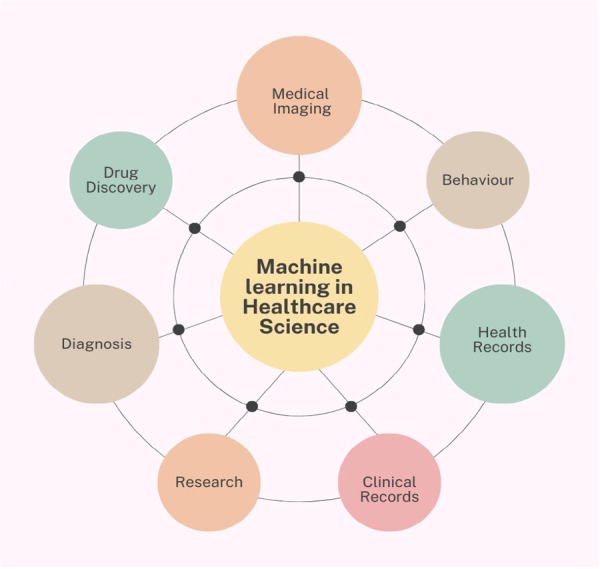

## 1 Introduction

### 1.1 Role of Artificial Intelligence in Multidrug‐Resistant Bacteria

**Figure 1:**
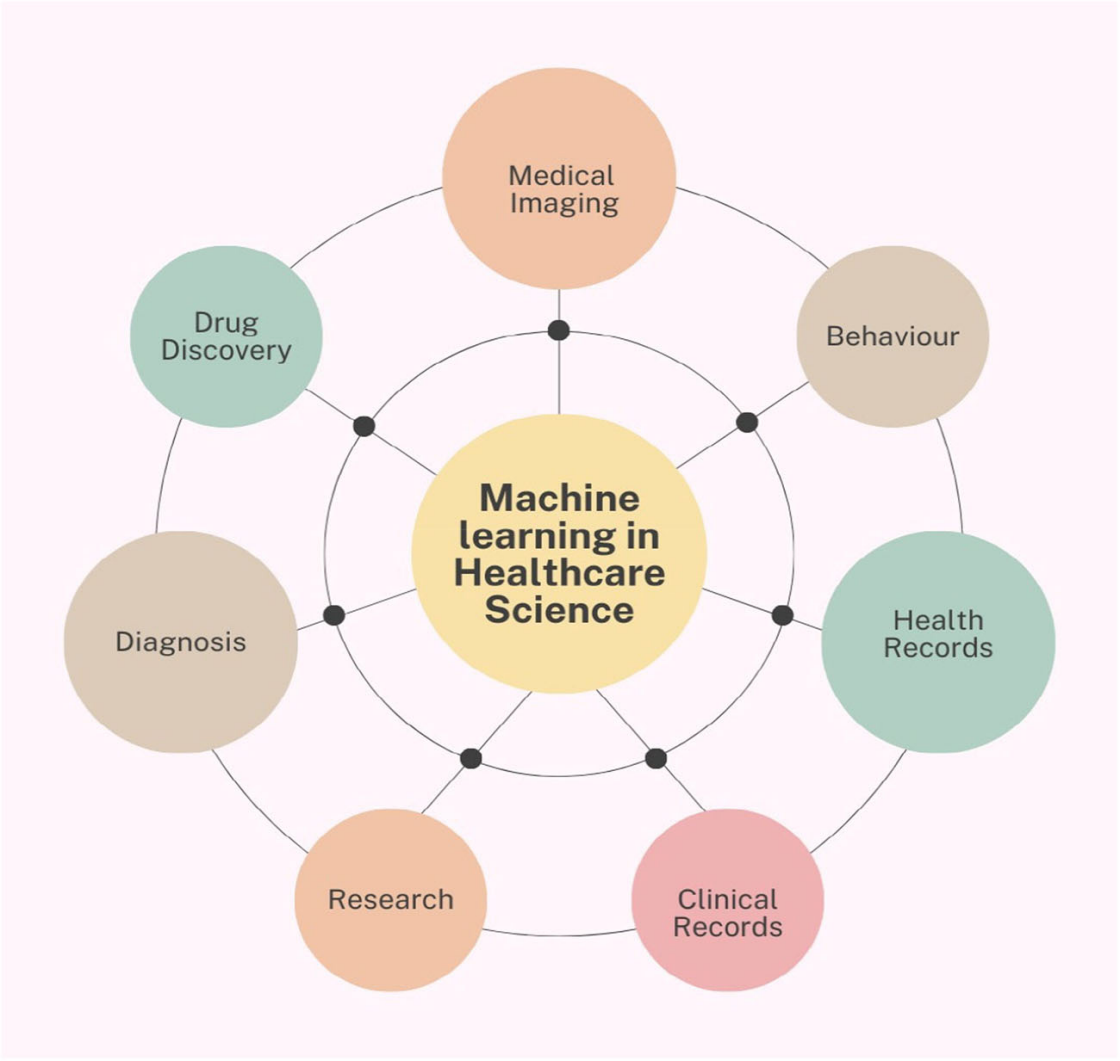
Application of machine learning in various healthcare sectors.

In clinical settings, the misuse of antibiotics has accelerated the development of bacteria resistant to medications, leading to the rapid spread of germs that are resistant even to the most powerful antibiotics. In 1928, Alexander Fleming discovered penicillin, an event widely regarded as the beginning of the modern era of antibiotic treatment. The discovery of antibiotics has provided significant benefits to persons afflicted by infections caused by bacteria and fungi. However, the overuse of antibiotics in hospitals has contributed to the development of drug resistance and the emergence of medication‐resistant germs (Bilal et al. 2021). As of the year 2050, it is estimated that the annual mortality rate associated with AMR will reach 10 million, potentially causing an economic impact of 100 billion dollars. Successfully combating the pandemic of antibiotic resistance requires the adoption of new regulations and renewed research efforts (Onifade et al. 2025). Artificial intelligence (AI) and machine learning are used across various sectors, but they have shown remarkable results in identifying multidrug‐resistant bacteria, as illustrated in Figure 1.

When compared to their Western counterparts, political leaders in Japan and China have placed greater emphasis on arguments that resonate more with the public. In 2016, the Chinese government launched a program called NAPCAR, an acronym for the National Action Plan to Combat AMR (Van Gemert 2017). It is of utmost importance to continue developing novel antimicrobial peptides (AMPs), antibiotic combinations, and monitoring technologies to combat AMR. This is done to prevent the spread of antibiotic‐resistant bacteria. Recent research has shown that AI is highly effective in minimizing antibiotic resistance. AMR can be studied through various approaches, one of which involves the use of AI technologies based on sequencing (Lv et al. 2021). Clinical decision support systems based on aggregated clinical data can likely assist practitioners in promoting antibiotic stewardship. These systems simplify the monitoring of changes in AMR (Lau et al. 2021). Furthermore, AI is frequently utilized in the discovery of new antibiotics and in studying the interactions between various medicinal combinations (Raisch and Krakowski 2021). The structural and molecular mechanisms underlying antibiotic resistance have been the primary focus of most research conducted to date (Song et al. 2019).

This review aims to explore how machine learning can be used to address the growing challenge of antibiotic resistance and to highlight its potential in shaping a new era of infectious disease control. Specifically, the review seeks to provide an overview of the burden of antibiotic resistance on global health, while examining how machine learning is being applied in areas such as detection, prediction, drug discovery, and treatment optimization.

### 1.2 The AMR Crisis

According to Cosgrove (2006), AMR is a significant concern in the modern public health system. It refers to the process by which bacteria and other pathogens develop mechanisms to evade the effectiveness of antimicrobial drugs over the course of their evolution (Ahmed et al. 2022; Cosgrove 2006). The occurrence of this condition elevates the probability of disease transmission, critical illness, and mortality. Additionally, it may become more difficult to treat infections effectively. The main cause of antibiotic resistance, which originates from the process of biological evolution, is the overuse of antimicrobials in agriculture, animal husbandry, and human health. According to Imran et al. (2022), AMR poses a significant threat to global public health, the stability of the global economy, and the ability of healthcare systems to deliver effective treatment in the future (Imran et al. 2022).

It is estimated that antibiotic‐resistant diseases cause approximately 700,000 deaths worldwide each year (O'Neill 2016). If the current trend continues, it is anticipated that this figure will reach 10 million by the year 2050. This significant economic issue may have a detrimental impact on various industries, including the food and agricultural sectors, the productivity sector, and the healthcare industry. Low‐income countries are particularly vulnerable to the effects of AMR due to inadequate healthcare infrastructure and a higher prevalence of infectious diseases. The acceleration of AMR can be attributed to several factors. The situation is further exacerbated by the fact that resistant strains can rapidly spread across continents and borders, facilitated by the ease of global travel and trade.

In addition, if AMR is not controlled, it has the potential to cause far‐reaching and devastating consequences for the ecosystem. AMR directly impacts individuals by increasing healthcare costs, prolonging hospital stays, and raising mortality rates. These are just some of the direct effects. According to White and Hughes, critical medical treatments such as organ transplants, chemotherapy, and major surgeries depend on the availability of effective antimicrobials to prevent infections. Since these procedures rely on infection prevention, AMR poses a significant threat to society (White and Hughes 2019). AMR makes it more difficult to prevent infectious disease outbreaks, as demonstrated by cases involving MRSA and multidrug‐resistant tuberculosis MDR‐TB. This is because AMR hinders the effective eradication of infectious diseases.

AMR is a critical issue that must be addressed comprehensively through an international effort involving multiple healthcare specialties. Previous strategies, no matter how extensive, have proven insufficient against the current wave of resistance. Therefore, there is an urgent need for new research exploring potential treatments, including the application of AI to improve antibiotic resistance management. Immediate investigation is essential. The incorporation of AI in combating the antibiotic resistance pandemic could open the door for innovative methods in research, diagnosis, treatment, and healthcare delivery on a global scale. The following sections will discuss these potential technological applications in detail.

## 2 Using AI to Combat Antibiotic Resistance

This study aims to use AI to better understand and fight antibiotic resistance. AI tools to predict resistance patterns, help doctors choose the right antibiotics, and identify new potential drugs to treat resistant infections. Antibiotic resistance has become increasingly widespread due to the overuse of antibiotics, leading to numerous undesirable consequences. As a result of AMR, antibiotics have become significantly less effective, which is a major concern (Arango‐Argoty et al. 2018). This has led to a significant decline in the efficacy of antibiotics (Cánovas‐Segura et al. 2016). Although WGS‐AST has demonstrated potential in detecting AMR, comprehensive data sets encompassing various characteristics are essential for efficient information extraction (Song et al. 2019). Significant progress in the state of the art has been achieved as a result, leading to the incorporation of AI into the previously described approaches (Lv et al. 2021).

One of the most important aspects of computer science is the study of AI, a fundamental component of the field. This area focuses on developing systems that emulate human cognition and is continually advancing. AI technology aims to enhance various activities, including visual perception, speech identification, processing of natural languages, and data‐driven decision‐making (Lv et al. 2021; Raisch and Krakowski 2021). The purpose of technology is to enhance human capabilities. A significant portion of the progress in AI systems depends on the availability of health data and advancements in computing power. The development of processing capacity and EHRs are two examples of areas that rely on complex mathematical techniques such as neural networks, machine learning, and deep learning (Ahmed et al. 2020). Both components depend on these approaches. The complexity of deep neural network topologies, which can be defined as the number of components the networks need to integrate, has increased dramatically over the past decade (Zafar et al. 2009). This is especially relevant at the present moment due to the significant increase in the number of components that networks are required to integrate.

When faced with massive data sets, the use of AI in ML allows for significant advancements (Nava Lara et al. 2019). Brain networks are models used in the field of numerical informatics. Their purpose is to approximate data representation in a way that mimics the functioning of real brain networks. These models can detect underlying connections within a data set by leveraging data linkages. In a deep neural network DNN, multiple layers of processing units are typically added, enabling researchers to analyze data independently and generate more accurate predictions based on the results (van Belkum et al. 2020). While neural networks can adapt to new input sources (Lv et al. 2021), the performance of neural networks can improve with a growing data set (Gajdács et al. 2020). Neural networks can also adapt to new input sources. The process of delivering medication has become more complex due to various factors related to a patient's preferences and clinical history. Based on a recent estimate (Lv et al. 2021), It is anticipated that the volume of clinical data available to the public this year will exceed what an average person could read in their entire lifetime. Due to its inherent ability to analyze massive data sets, AI is expected to play a significant role in clinical operations. However, the vast majority of professionals still rely on intuitive judgment and established medical norms rather than AI when making evaluations (Song et al. 2019). The objective of this audit is to demonstrate how AI can help reduce the growing problem of antibiotic resistance in pediatric patients. The primary focus was on the use of AI in treating pediatric illnesses in industrialized countries.

### 2.1 AI‐Powered Approaches to Managing AMR

This study aims to explore how AI can help manage AMR. It will use AI to track resistance trends, improve antibiotic use, and support the discovery of new treatments to reduce the impact of resistant infections. The use of AI in AMR control systems represents an innovative approach to addressing this global health threat. AI could lead to improvements in AMR diagnosis, treatment, and prevention in various ways, including the discovery of novel drugs and the enhancement of patient care (Tran et al. 2022). All these improvements may be achieved through the utilization of AI. This section aims to highlight the potential for AI to transform AMR activities and to explore the future of healthcare solutions. Our goal is to emphasize how AI can revolutionize AMR efforts.

### 2.2 AI in the Development and Discovery of Antibiotics

This study aims to use AI to speed up the discovery and development of new antibiotics. It will apply AI to analyze large data sets, predict effective drug compounds, and identify novel molecules that can fight resistant bacteria. The purpose of algorithms driven by AI is to evaluate vast chemical libraries and predict compounds that may exhibit antibacterial properties. This significantly accelerates the early stages of the drug development process. For example, deep‐learning algorithms can identify molecular structural features associated with antibiotic activity, helping to narrow the search for viable candidates with the potential to combat bacteria resistant to existing therapies (Lau et al. 2021). AI holds promise for enhancing the design of clinical trials for novel antibiotics by discerning the most promising drug candidates, forecasting patient reactions to treatment, and guaranteeing a varied participant pool (Rabaan et al. 2022). This can be achieved by evaluating which drug candidates show the most promise. Consequently, this approach not only reduces the time and costs associated with trials but also increases the likelihood of successfully discovering practical new treatments.

One of the earliest successes was halicin, discovered at MIT in 2020 using a deep‐learning model trained on chemical and antibacterial activity data. The AI predicted halicin, a compound originally developed for diabetes, as a potent antibiotic. Experiments showed it was active against a broad range of pathogens, including pan‐resistant *Acinetobacter baumannii* and *Clostridioides difficile*. In mouse infection models, halicin sterilized *C. difficile* infections and healed wounds infected with resistant *A. baumannii*. Remarkably, bacteria did not quickly develop resistance even after prolonged exposure, unlike with conventional antibiotics such as ciprofloxacin. Around the same time, researchers also discovered abaucin, a compound selectively targeting *A. baumannii*. Unlike broad‐spectrum drugs, abaucin was narrow‐spectrum, sparing most other species (Awan et al. 2024). It interfered with the LolE lipoprotein translocation pathway and greatly decreased the bacterial burden in mouse wound infections. These two findings demonstrated that AI was able to provide not only a wide‐spectrum but also precision antibiotics (Song and Shim 2025).

The AI models have since been implemented on even greater scales. The AMPSphere initiative was a metagenomics and genomic mining of the global microbiome, which predicted close to 1 million AMPs (Santos‐Júnior et al. 2024). A large number of synthesized candidates demonstrated high activity toward resistant pathogens in vitro, with some of them demonstrating activity in animal infection models. Correspondingly, scientists at MIT used machine learning to screen 12 million molecules against MRSA and found some compounds that decreased the bacterial load by tenfold in mouse skin and systemic infections.

### 2.3 AI in Susceptibility Testing and Diagnostics

This study aims to use AI to improve antibiotic susceptibility testing and diagnostics. It will focus on developing AI tools that quickly and accurately detect resistant bacteria, helping clinicians choose the most effective treatment. To achieve rapid pathogen identification from clinical samples, machine‐learning methods in conjunction with genetic sequencing and analysis are used in AI‐enhanced diagnostics (Melo et al. 2021). This approach is implemented to achieve rapid pathogen identification. With access to early detection, clinical practitioners can make better‐informed decisions regarding the treatment of illnesses where every second counts. The use of machine‐learning algorithms enables the analysis of past patterns of antibiotic resistance to predict which diseases will likely tolerate certain treatments. According to McCarlie et al. this predictive capability facilitates the development of more precise and personalized treatment regimens, potentially reducing the need for broad‐spectrum antibiotics and thereby slowing the rise of resistance (Mc Carlie et al. 2022).

### 2.4 Support Vector Machines (SVMs)

β‐lactams are the most commonly used class of antibiotics in clinical settings. Consequently, it is unsurprising that β‐lactamases represent the most prevalent mechanism by which bacteria develop resistance to these antibiotics. Over time, various chemical modifications of β‐lactams have been created, which in turn have driven the evolution of bacterial β‐lactamases. These enzymes degrade β‐lactam antibiotics, rendering them ineffective. Therefore, accurately identifying β‐lactamases is critically important to ensure appropriate therapeutic interventions. To streamline this laborious and time‐consuming laboratory process, a model utilizing SVM techniques was developed (Srivastava et al. 2018). This model has been developed to simplify operations. It is a classification method well‐known in the field of machine learning for its robustness against outliers and its effectiveness in processing high‐dimensional data sets, which are commonly encountered in bioinformatics (Van Messem 2020). The SVM is a classification method known for its robustness against outliers. Multi‐level SVM models are employed to classify β‐lactamase enzymes into categories A, B, C, or D. According to Chou (2001, 2005), these models are based on protein sequences characterized by either amino acid composition (AAC) or pseudo amino acid composition PseAAC. Additionally, category B can be further subdivided into B1, B2, and B3. Models using PseAAC as input demonstrated strong performance, achieving accuracy values ranging from 82% to 97%. Leave‐One‐Out Cross‐Validation (LOOCV) was used to validate these models (Chou 2001, 2005). According to further studies conducted by the Srivastava group, SVM models were employed to investigate efflux pump proteins, which represent an additional mechanism of antibiotic resistance. BacEffluxPred is a dual‐tier ensemble of SVM models were created to recognize and categorize bacterial efflux pump proteins into distinct groups, as reported by Pandey et al. in 2020 (Pandey et al. 2020). The model I developed to identify antibiotic resistance efflux ARE proteins from non‐ARE proteins achieved an accuracy of 85% on the training data set and 94% on an independent data set. Both results are impressive. Using LOOCV, the Level II model was tested by comparing each ARE category, ABC transporters, MFS, SMR, and MATE against the other three combined. The model achieved about 93% accuracy for the first three groups, while MATE reached a perfect score of 100%. These results were compared to those of the combined group of the other three classes. Furthermore, on two independently selected data sets, a SVM model obtained accuracies of 79% and 72% (Gupta et al. 2022). This was achieved by using sequences characterized by the frequency of dipeptides and pepstatin‐containing vectors. As a result, the model demonstrated greater efficiency in distinguishing between harmful and non‐pathogenic bacterial proteins. Toxins and antibiotic resistance genes were included as two of the three categories used to classify pathogenic proteins, as shown in Table 1.

**Table 1:** Various machine‐learning algorithms used in the antibiotic's resistance of various pathogen.

Authors	Year	Geographical setting	Data source	Algorithms used	Model performance	Target bacteria
Goodman et al. [34]	2016	USA	Blood culture results and AST profiles	Recursive partitioning, Decision Trees	Predictive values around 91% (PPV) and 92% (NPV)	*Escherichia coli, Klebsiella pneumoniae, Klebsiella oxytoca*
Vazquez‐Guillamet et al. [35]	2017	USA	EHR data combined with cultures and AST	Recursive partitioning, Decision Trees	AUC values ranged from 0.61 to 0.80	Gram‐negative bacilli
Sousa et al. [36]	2019	Spain	Clinical and demographic information with culture/AST results	Decision Trees	AUC 0.76	β‐lactam resistant GNB
Moran et al. [37]	2020	UK	Blood and Urine Cultures	XGBoost	AUC around 0.70	*E coli, Klebsiella pneumoniae, Pseudomonas aeruginosa*
Feretzakis et al. [38]	2020	Greece	Demographics/Cultures/AST/Bacterial Gram stain/Sample type	Multiple logistic regression	AUC 0.758	All isolated bacterial species
Feretzakis et al. [39]	2020	Greece	Demographics/Cultures/AST/Gram stain/Sample type	LR, RF, k‐NN, J48, MLP	AUC 0.726	All isolated bacterial species
Feretzakis et al. [40]	2021	Greece	Demographic and laboratory data with AST and Gram stain	JRip, RF, MLP, Class, Regression class, REPTree	F‐measure ~0.88, AUC up to 0.93	*P aeruginosa, Acinetobacter baumannii, K pneumoniae*
Martínez‐Agüero et al. [41]	2019	Spain	Demographics/Clinical Data/Type of Sample/Cultures/AST	LR, k‐NN, DT, RF, MLP	Accuracy for quinolone resistance 88.1 ± 1.6	*Pseudomonas, Stenotrophomonas, Enterococcus*
McGuire et al. [42]	2021	USA	Demographic, Medication, Vital Sign, Laboratory, Billing Code, Procedure, Culture, Sensitivity Data (67 features)	XGBoost	AUC 0.846	Carbapenem‐resistant isolates
Pascual‐Sánchez et al. [43]	2021	Spain	Electronic health record data	LR, DT, RF, XGBoost, MLP	AUC 0.76	MDR bacteria
Garcia‐Vidal et al. [44]	2021	Spain	Electronic health record data sets	RF, GBM, XGBoost, GLM	AUC 0.79	MDR‐*Pseudomonas aeruginosa*/ESBL‐E
Henderson et al. [45]	2022	USA	Electronic health record information	Penalized LR, Naïve Bayes, Gradient Boosting, SVM, RF	AUC 0.70	MDR‐*E. coli*

**Figure 2:**
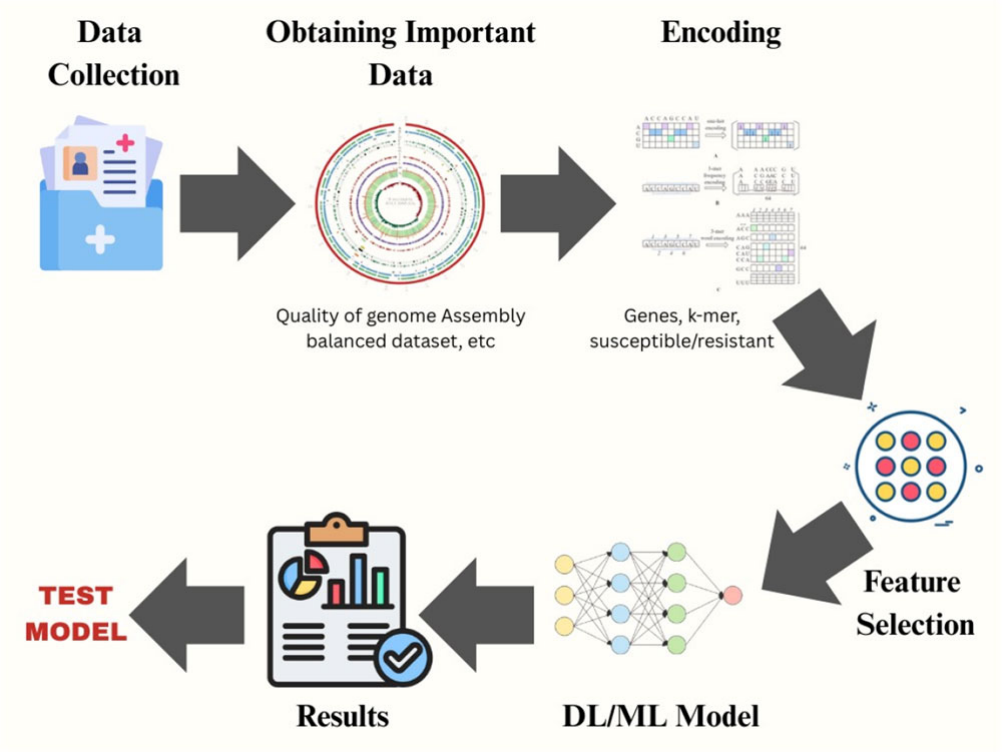
Testing of the machine‐learning model by providing data in sequences to obtain the required results.

Within the realm of deep‐learning‐based methods for identifying ARGs ARGs, DeepARG stands out as the most notable approach. DeepARG consists of two distinct artificial neural network ANN models: DeepARG‐LS, which is designed to detect ARGs from assembled sequences, and DeepARG‐SS, which is used for short reads (Arango‐Argoty et al. 2018). After being trained on DeepARG‐DB, which is a database that has been meticulously curated and obtains its information from UniProt (Apweiler 2004), Comprehensive Antibiotic Resistance Database, CARD (Jia et al. 2017), and ARDB (Liu and Pop 2009). The models achieved accuracy and recall scores of greater than 90% and 97%, respectively, demonstrating their strong ability to accurately identify relevant information. The data were organized into *N* × 4333 vector matrices representing bit scores. These matrices included UniProt training sequences as well as existing sequences from CARD and ARDB, all structured in this format. The bit scores quantified the similarity between the two sets of sequences and were aggregated accordingly. Subsequently, the protein sequences considered for model training were organized into these matrices. The matrices were then analyzed using artificial neural networks ANNs, enabling the prediction of 30 different categories of antibiotic resistance, including an “unknown” category. Additionally, the matrices underwent further analysis using the large language model LLM, ESM‐1b, which was initially trained on approximately 250 million protein sequences. This model was combined with XGBoost to detect ARGs and classify their corresponding resistance groups, thereby achieving the stated objectives (Rives et al. 2021). To identify and classify ARGs, PLM‐ARG utilized ESM‐1b to embed protein sequences. Subsequently, XGBoost models were trained on these embeddings to perform the task (Wu et al. 2023). The authors made this discovery through their research. They compared the performance of three prominent ARG prediction algorithms, RGI, ResFam, and DeepARG, with that of PLM‐ARG, which demonstrated superior results. In an independent test data set, PLM‐ARG achieved AUC metrics ranging from 9.6% to 36% and F1‐scores ranging from 40.8% to 107.3%. These results were obtained using the learning algorithm with various types of input data to evaluate the machine‐learning model, as illustrated in Figure 2.

Several distinct types of ARGs have been created to enhance our understanding of the propagation of antibiotic resistance and its associated ecological impacts. Li et al. introduced the concept of HMD‐ARG, an acronym for Hierarchical Multi‐task Deep‐learning framework, designed for ARG annotation (Li et al. 2021). This framework employs multiple distinct tiers for classifying ARGs and is based on Convolutional Neural Networks CNN. The model analyses a raw protein sequence encoded using one‐hot encoding and then proceeds through a series of predictions. These predictions include determining whether the sequence is an ARG, identifying the associated antibiotic resistance class, elucidating the resistance mechanism, distinguishing whether the ARG is acquired or intrinsic, and, if applicable, identifying the subclass of β‐lactamase. The sequences are meticulously curated from seven databases, including the CARD, a curated repository of known ARGs and associated mutations. CARD contains detailed information on resistance mechanisms, gene sequences, and phenotypic data (Jia et al. 2017), AMR Finder, or AMR Finder, is a tool used to identify AMR genes, resistance‐associated point mutations, and virulence genes in bacterial genomes (Feldgarden et al. 2019), ResFinder (Detects acquired ARGs in bacterial whole‐genome sequences WGS data) (Zankari et al. 2012), ARG‐ANNOT is a database and annotation tool for ARGs. It focuses on manually curated reference sequences of known resistance genes (Gupta et al. 2014) Deep ARG, A deep learning–based tool for ARG prediction. Uses neural networks trained on known ARGs to improve sensitivity and reduce false positives (Arango‐Argoty et al. 2018), MEGA Res (A large, structured database of ARGs designed for high‐throughput metagenomic sequencing data) (Lakin et al. 2019) and Resfams Uses profile hidden Markov models HMMs to detect more distant homologs than simple BLAST searches) (Gibson et al. 2015), were organized into 15 antibiotic resistance categories and six resistance mechanisms, including enzyme inactivation, target modification, plasmid‐mediated resistance, cell wall or membrane alterations, efflux pump upregulation, and mutation‐driven resistance, with an additional evaluation of gene transferability. The validation of the HMD‐ARG model's predictions was conducted through experimental testing of eight randomly selected genes from *Pseudomonas aeruginosa*. Each gene received an accurate score exceeding 0.9. In the field of ARG classification, one effective approach is an ensemble method called ARG‐CNN, which utilizes a convolutional neural network “CNN” for classifying sequence embeddings. Another technique, ARG‐InterPro, employs logistic regression to classify data related to protein domains, families, and functional locations. Lastly, the ARG‐KNN approach uses k‐nearest neighbor “KNN” classification of BLAST alignment homology data to categorize ARGs (Wang et al. 2021). There are three different approaches to categorizing ARGs. These methods can be compared to several previously published ARG classification techniques, such as the DIAMOND best‐hit approach. (Buchfink et al. 2015), BLAST best hit (Altschul et al. 1990), TRAC (Hamid 2019), DeepARG (Arango‐Argoty et al. 2018), and HMMER (Eddy 2011), the entire model, named ARG‐SHINE, was able to outperform previously published methods.

For the purpose of highlighting articles for human assessment, text‐mining techniques were applied to ARG databases that had been manually curated, such as CARD (Jia et al. 2017). Incorporating deep‐learning into this process through natural language processing has the potential to drive further breakthroughs in language comprehension. To predict the relationships between genes and antibiotics, a Biomedical Relation Extraction BioRE method was developed. This system was trained at the phrase level using the data sets of PubMed, CARD (Jia et al. 2017), and UniProtKB (Apweiler 2004). They were all used for training the system. This approach was designed with the intention of enhancing the curation of ARGs from published works (Brincat and Hofmann 2022). Separate training sessions were conducted on the data sets for two models: the transformer‐based BioBERT, which utilizes biological data, and the piecewise convolutional neural network (PCNN). These data sets served as the training data for both models. BioBERT was utilized to find metronidazole gene‐antibiotic correlations in *Helicobacter pylori* after demonstrating exceptional performance on the holdout test data set.

### 2.5 Hidden Markov Models (HMM)

HMMs were created for each gene cluster to build multiple HMM models that recognize an input sequence as an ARG (Arango‐Argoty et al.) and forecast its source (Lakin et al. 2019). These models are well‐known for their ability to predict the source of ARGs. To develop these models, genes from the MEGARes database were utilized (Lakin et al. 2017) were utilized and categorized according to the degree of sequence similarity among them. The model achieved mean specificity and sensitivity values ranging from 97% to 99%, indicating that both metrics are very high. HMMs based on these indicators were used by Xie and Fair (Xie and Fair 2021) to accurately identify virulence factors, antibiotic resistance sequences and bacterial toxins from next‐generation sequencing (NGS) data, the integrated the Short Better Representative Extract Data set's ShortBRED junction marker features with distinctive family‐specific substring markers.

### 2.6 Challenges and Limitations

AI has the potential to deliver revolutionary results in the battle against AMR, but its application in health care and research faces several barriers and limitations (Tran et al. 2022). Although AI has the potential to deliver revolutionary results, it is necessary to address a variety of challenges to fully harness the power of AI in managing AMR. These challenges include technical, ethical, data‐related, and structural issues. Medical professionals and researchers may find it difficult to understand the decision‐making processes of AI systems, especially deep‐learning models (Song et al. 2023). The reason for this is the presence of a technical barrier. In the context of executing therapeutic processes, the “black box” problem affects the ability to understand and trust the insights provided by artificial intelligence, which is crucial for the success of these procedures. Significant technical challenges must be overcome when integrating AI technology into existing healthcare infrastructures (Dennehy et al. 2023), providing dependable performance in a variety of healthcare environments, managing the computational resources needed for AI applications, and being compatible with electronic health record (EHR) systems. According to Thakur et al.(2022), one of the most important factors to consider when measuring the effectiveness of AI models is the availability of data sets that are both extensive and of high quality (Thakur et al. 2022). In addition, there are ongoing issues with the data. Privacy concerns, inconsistent data sources, and the underrepresentation of particular groups or situations can all make it more difficult to collect this kind of data, which can lead to biased or incomplete models. These factors further exacerbate the problem of underrepresentation. According to Chen and Esmaeilzadeh, there are serious privacy invasion and security protection concerns when sensitive patient data is used in AI algorithms (Chen and Esmaeilzadeh 2024). It is essential to implement robust data security measures, which can be challenging and costly, to ensure the safety and integrity of health data used by applications employing artificial intelligence. This is essential to preserving trust and preventing data leaks. It is important to note that these issues add to the social and ethical challenges previously discussed. To successfully address these difficulties, it is necessary to develop ethical standards that are transparent and subject to oversight. Establishing public trust in healthcare solutions powered by AI is necessary for their broad acceptance. This is necessary to address concerns and questions regarding artificial intelligence. According to Alami et al.'s (2020) research, obtaining regulatory approval for AI‐based healthcare innovations can be time‐consuming and challenging, with significant variations across different countries (Alami et al. 2020). Developing standards that promote innovation while ensuring the safety and efficiency of applications utilizing AI is a critical challenge that must be addressed. According to Ahmad et al.'s (2020) research, effectively managing antibiotic resistance with AI requires collaboration across multiple disciplines (Ahmad et al. 2020). These fields include microbiology, pharmacology, computer science, and healthcare policy. However, promoting interdisciplinary cooperation presents challenges due to differences in language, techniques, and objectives. Despite these obstacles, they can be overcome.

Utilizing AI to fight against AMR presents a variety of significant challenges and limitations, including technical, ethical, data‐related, and systemic issues (Ali et al. 2023). Each of these challenges and limitations is equally important. Successfully addressing them requires a collaborative effort involving academics, healthcare practitioners, legislators, and technology entrepreneurs. Establishing open and transparent ethical standards, efficient data governance frameworks, and interoperable healthcare technologies will facilitate finding solutions to the healthcare industry's difficulties. Furthermore, to fully harness the potential of AI in combating AMR, it is essential to promote interdisciplinary collaboration and engage the public to build trust. This approach is vital to maximizing AI's impact.

Despite these challenges, AI offers significant potential to enhance methods for combating antibiotic resistance. The global healthcare community can promote innovative and effective solutions to one of the most pressing health challenges of our time if it acknowledges and actively addresses these difficulties. This is the situation we currently face.

### 2.7 Policy and Regulatory Considerations

When considering how AI can be integrated into AMR projects, it is essential to account not only for the scientific and technical capabilities of technology but also for the complex regulatory and policy frameworks. AI has the potential to be significantly more effective than traditional methods. Given the crucial role these frameworks play, it is possible to develop AI applications that are efficient, safe, moral, and egalitarian. According to the World Health Organization, supporting the implementation of policies by national and international organizations is vital to promote the development and use of AI technology in the healthcare sector (Guidance 2021). Collaborative efforts between the public and corporate sectors, financial incentives for discovering new antibiotics, and similar initiatives exemplify this approach. Developing standardized protocols for the collection, dissemination, and analysis of data is essential to maximize the effectiveness of AI systems across various healthcare settings and to ensure their interoperability. For policymakers to contribute effectively to the global fight against AMR, advocating for the establishment of universal health data standards is crucial. It is important to recognize the need for access to large, high‐quality data sets to successfully train and validate AI models. Current regulations support this objective. Two viable strategies include creating secure pathways for data exchange and implementing systems to obtain patients' consent for the appropriate use of their medical records (Dwivedi et al. 2021). Both of these methods are examples of potentially effective approaches.

In addition, regulatory agencies are required to review and approve any medical software or equipment based on artificial intelligence. According to Sun and Medaglia, the primary goals for the acceptance criteria of AI applications should focus on clinical validation, transparency, and accountability, as these are the three most critical aspects of the process (Sun and Medaglia 2019). As AI continues to grow rapidly and models evolve dynamically, traditional regulatory procedures are facing significant challenges. The continuous development of AI technology necessitates that regulatory bodies adopt innovative approaches, such as adaptive regulation. Given that AMR poses a global threat, it is crucial for governments to collaborate in establishing unified regulatory standards. According to Galindo et al. harmonizing regulations across different countries is essential to facilitate the worldwide deployment of AI systems (Galindo et al. 2021). In the context of AI, legislators face a wide range of ethical challenges. These include concerns about bias, lack of transparency, and the potential loss of jobs in the healthcare sector. Establishing ethical guidelines and standards for the use of AI in medicine could help mitigate some of these risks. AI‐powered AMR strategies should be made accessible to everyone, especially in resource‐limited regions. Policymakers are responsible for ensuring this accessibility. Efforts are underway to reduce disparities in healthcare systems, data representation, and access to technological resources (Cihon 2019). This is a component of the overall endeavor.

In addition, multidisciplinary teams are essential for effectively integrating AI into efforts to control antibiotic resistance within the healthcare industry. Governments must prioritize the promotion of interdisciplinary research teams. These teams should include microbiologists, physicians, AI developers, and ethicists. Providing accurate information to policymakers, the general public, and healthcare practitioners about the benefits and challenges of AI in healthcare is crucial. Moonesar and Dass as well as Reddy et al. have proposed that healthcare education programs incorporating data science and AI could help achieve this goal (Moonesar and Dass 2021; Reddy et al. 2020). Additionally, opportunities for ongoing professional growth should be pursued throughout this process. It is essential to address a broad array of legal and regulatory concerns to ensure that AI is appropriately integrated into strategies for controlling AMR. To effectively combat the pandemic of AMR and ensure a safer, healthier future for people worldwide, the legislative process must adopt a proactive and innovative approach to drafting laws and policies. The importance of this cannot be overstated, especially in light of recent advances in AI technology. Such frameworks are necessary to promote innovation, safety, efficacy, and collaboration across various disciplines, while also addressing ethical and equitable considerations.

## 3 Future Perspective

Moving forward, machine learning is likely to become a crucial component of infectious disease research in ways that are not yet fully apparent. Machine learning will not only assist in discovering new antibiotics as computational power and data become more diverse, but it will also inform personalized treatment plans, optimize antibiotic stewardship initiatives, and enhance real‐time monitoring of resistance dynamics (Kumari et al. 2025). The next phase will not focus on whether AI can work, but rather on how to utilize it responsibly and at scale to achieve tangible improvements in global health. One of the most promising directions is the integration of machine learning with high‐throughput experimental platforms, such as automated wet‐lab validation and robotics, to rapidly transition from digital predictions to real‐world testing (Palanikumar et al. 2025). Another exciting avenue is the application of generative AI to design entirely new classes of antibiotics and AMP, as early studies on halicin and abaucin have demonstrated. Integration with global genomic surveillance networks also holds tremendous potential, machine learning could detect emerging resistance patterns in real time, enabling health systems to act before outbreaks escalate into crises (Wei et al. 2025). Despite rapid progress, several key questions remain unresolved. Finally, there is the broader issue of access, ensuring that these innovations benefit not only well‐resourced laboratories but also low‐ and middle‐income countries where antibiotic resistance is most severe. Addressing these unanswered questions will determine whether machine learning truly fulfills its promise in the fight against AMR (Seele 2024).

## 4 Conclusion

In the ongoing battle against AMR, research into AI is proving to be highly valuable. Researchers have concluded that AI offers innovative tools with the potential to improve traditional methods of controlling AMR. AI has the capacity to revolutionize various domains, including the development of antibiotics, diagnostics, treatment regimens, and surveillance systems. For example, AI can accelerate and enhance the accuracy of antibiotic discovery. Therefore, AI can revitalize the antibiotic research pipeline, which has remained stagnant for a considerable time without significant advancements. Moreover, AI holds great promise for reducing antibiotic use and resistance by improving the speed and accuracy of disease diagnosis and predicting antibiotic resistance profiles. Its ability to analyze vast amounts of data can be advantageous for monitoring and preventing AMR, benefiting both policymaking and public health efforts. However, integrating AI into AMR strategies presents challenges, including technological complexity, ethical concerns, the need for robust legal frameworks, and data protection requirements. Additionally, effective AI solutions require collaboration among healthcare practitioners, researchers, and policymakers across various sectors, along with ongoing education and capacity building. To ensure AI contributes effectively to the fight against AMR, it is essential to address these challenges collaboratively. Developing ethical, open‐source, and transparent AI models is crucial for global impact, especially in resource‐limited countries where antibiotic resistance is a critical issue. With AI's support in predicting, preventing, and treating infections caused by antibiotic‐resistant bacteria, significant progress in combating this growing threat is achievable. However, to make this promise a reality, the scientific community, the healthcare industry, and the government will need to collaborate to foster innovation while addressing the legal, ethical, and technical challenges associated with AI applications.

## Author Contributions

**Akmal Zubair:** conceptualization, writing – original draft, investigation, formal analysis. **Mohd Fazil:** writing – review and editing. **Muhammad Jawad:** conceptualization, investigation, formal analysis. **Safa Wdidi:** upervision, writing – original draft, writing – review and editing.

## Conflicts of Interest

None declared.

## Data Availability

The authors have nothing to report.

## References

[mbo370160-bib-0001] Ahmad, O. F. , D. Stoyanov , and L. B. Lovat . 2020. “Barriers and Pitfalls for Artificial Intelligence in Gastroenterology: Ethical and Regulatory Issues.” Techniques and Innovations in Gastrointestinal Endoscopy 22, no. 2: 80–84.

[mbo370160-bib-0002] Ahmed, I. , M. B. Rabbi , and S. Sultana . 2019. “Antibiotic Resistance in Bangladesh: A Systematic Review.” International Journal of Infectious Diseases 80: 54–61. 10.1016/j.ijid.2018.12.017. 30634043

[mbo370160-bib-0004] Ahmed, N. , M. Khan , W. Saleem , et al. 2022. “Evaluation of Bi‐Lateral Co‐Infections and Antibiotic Resistance Rates Among COVID‐19 Patients.” Antibiotics (USSR) 11, no. 2: 276. 10.3390/antibiotics11020276 PMC8868529 35203877

[mbo370160-bib-0003] Ahmed, N. , Z. Ali , M. Riaz , B. Zeshan , J. I. Wattoo , and M. N. Aslam . 2020. “Evaluation of Antibiotic Resistance and Virulence Genes Among Clinical Isolates of Pseudomonas Aeruginosa From Cancer Patients.” Asian Pacific Journal of Cancer Prevention 21, no. 5: 1333–1338. 32458641 10.31557/APJCP.2020.21.5.1333 PMC7541853

[mbo370160-bib-0006] Alami, H. , P. Lehoux , Y. Auclair , et al. 2020. “Artificial Intelligence and Health Technology Assessment: Anticipating a New Level of Complexity.” Journal of Medical Internet Research 22, no. 7: e17707. 32406850 10.2196/17707 PMC7380986

[mbo370160-bib-0007] Ali, T. , S. Ahmed , and M. Aslam . 2023. “Artificial Intelligence for Antimicrobial Resistance Prediction: Challenges and Opportunities Towards Practical Implementation.” Antibiotics (USSR) 12, no. 3: 523. 10.3390/antibiotics12030523 PMC10044311 36978390

[mbo370160-bib-0008] Altschul, S. F. , W. Gish , W. Miller , E. W. Myers , and D. J. Lipman . 1990. “Basic Local Alignment Search Tool.” Journal of Molecular Biology 215, no. 3: 403–410. 2231712 10.1016/S0022-2836(05)80360-2

[mbo370160-bib-0009] Apweiler, R. 2004. “Uniprot: The Universal Protein Knowledgebase.” Supplement, Nucleic Acids Research 32, no. suppl_1: 115D–119D. 14681372 10.1093/nar/gkh131 PMC308865

[mbo370160-bib-0010] Arango‐Argoty, G. , E. Garner , A. Pruden , L. S. Heath , P. Vikesland , and L. Zhang . 2018. “Deeparg: A Deep Learning Approach for Predicting Antibiotic Resistance Genes From Metagenomic Data.” Microbiome 6: 23. 29391044 10.1186/s40168-018-0401-z PMC5796597

[mbo370160-bib-0011] Awan, R. E. , S. Zainab , F. J. Yousuf , and S. Mughal . 2024. “Ai‐Driven Drug Discovery: Exploring Abaucin as a Promising Treatment Against Multidrug‐Resistant Acinetobacter Baumannii.” Health Science Reports 7, no. 6: e2150. 10.1002/hsr2.2150. 38841115 PMC11150274

[mbo370160-bib-0014] van Belkum, A. , C.‐A. D. Burnham , J. W. A. Rossen , F. Mallard , O. Rochas , and W. M. Dunne, Jr. . 2020. “Innovative and Rapid Antimicrobial Susceptibility Testing Systems.” Nature Reviews Microbiology 18, no. 5: 299–311. 32055026 10.1038/s41579-020-0327-x

[mbo370160-bib-0015] Bilal, H. , M. N. Khan , T. Rehman , M. F. Hameed , and X. Yang . 2021. “Antibiotic Resistance in Pakistan: A Systematic Review of Past Decade.” BMC Infectious Diseases 21, no. 1: 244. 10.1186/s12879-021-05906-1. 33676421 PMC7937258

[mbo370160-bib-0016] Brincat, A. , and M. Hofmann . 2022. “Automated Extraction of Genes Associated With Antibiotic Resistance From the Biomedical Literature.” Database 2022: baab077. 35134132 10.1093/database/baab077 PMC9263533

[mbo370160-bib-0017] Buchfink, B. , C. Xie , and D. H. Huson . 2015. “Fast and Sensitive Protein Alignment Using Diamond.” Nature Methods 12, no. 1: 59–60. 25402007 10.1038/nmeth.3176

[mbo370160-bib-0018] Cánovas‐Segura, B. , M. Campos , A. Morales , J. M. Juarez , and F. Palacios . 2016. “Development of a Clinical Decision Support System for Antibiotic Management in a Hospital Environment.” Progress in artificial intelligence 5: 181–197.

[mbo370160-bib-0019] Mc Carlie, S. , G. Staats , B. Belter , B. Van Der Walt , and R. Bragg . 2022. Molecular Tools for the Study of Resistance to Disinfectants.

[mbo370160-bib-0020] Chen, Y. , and P. Esmaeilzadeh . 2024. “Generative AI in Medical Practice: In‐Depth Exploration of Privacy and Security Challenges.” Journal of Medical Internet Research 26: e53008. 38457208 10.2196/53008 PMC10960211

[mbo370160-bib-0021] Chou, K.‐C. 2001. “Prediction of Signal Peptides Using Scaled Window.” Peptides 22, no. 12: 1973–1979. 11786179 10.1016/s0196-9781(01)00540-x

[mbo370160-bib-0022] Chou, K.‐C. 2005. “Using Amphiphilic Pseudo Amino Acid Composition to Predict Enzyme Subfamily Classes.” Bioinformatics 21, no. 1: 10–19. 15308540 10.1093/bioinformatics/bth466

[mbo370160-bib-0024] Cihon, P. 2019. “Standards for Ai Governance: International Standards to Enable Global Coordination in AI Research & Development.” Future of Humanity Institute. University of Oxford 40, no. 3: 340–342.

[mbo370160-bib-0025] Cosgrove, S. E. 2006. “The Relationship Between Antimicrobial Resistance and Patient Outcomes: Mortality, Length of Hospital Stay, and Health Care Costs.” Supplement, Clinical Infectious Diseases 42, no. Suppl_2: S82–S89. 16355321 10.1086/499406

[mbo370160-bib-0026] Dennehy, D. , A. Griva , N. Pouloudi , Y. K. Dwivedi , M. Mäntymäki , and I. O. Pappas . 2023. “Artificial Intelligence (AI) and Information Systems: Perspectives to Responsible Ai.” Information Systems Frontiers 25, no. 1: 1–7.

[mbo370160-bib-0027] Dwivedi, Y. K. , L. Hughes , E. Ismagilova , et al. 2021. “Artificial Intelligence (AI): Multidisciplinary Perspectives on Emerging Challenges, Opportunities, and Agenda for Research, Practice and Policy.” International journal of information management 57: 101994.

[mbo370160-bib-0028] Eddy, S. R. 2011. “Accelerated Profile HMM Searches.” PLoS Computational Biology 7, no. 10: e1002195. 22039361 10.1371/journal.pcbi.1002195 PMC3197634

[mbo370160-bib-0030] Feldgarden, M. , V. Brover , D. H. Haft , et al. 2019. “Validating the Amrfinder Tool and Resistance Gene Database by Using Antimicrobial Resistance Genotype‐Phenotype Correlations in a Collection of Isolates.” Antimicrobial Agents and Chemotherapy 63, no. 11: e00483‐19. 10.1128/aac.00483-00419. 31427293 PMC6811410

[mbo370160-bib-0030a] Erratum in: *Antimicrobial Agents and Chemotherapy*. 2020 Mar 24; 64, no. 4: e00361‐20. 10.1128/AAC.00361-20.

[mbo370160-bib-0031] Gajdács, M. , E. Paulik , and A. Szabó . 2020. “Knowledge, Attitude and Practice of Community Pharmacists Regarding Antibiotic use and Infectious Diseases: A Cross‐Sectional Survey in Hungary (KAPPha‐HU).” Antibiotics (USSR) 9, no. 2: 41. 10.3390/antibiotics9020041 PMC7168197 31973119

[mbo370160-bib-0032] Galindo, L. , K. Perset , and F. Sheeka . 2021. “An Overview of National AI Strategies and Policies.” OECD Going Digital Toolkit Notes, 14. 10.1787/c05140d9-en.

[mbo370160-bib-0033] Van Gemert, T. 2017. On the Influence of Dataset Characteristics on Classifier Performance. Utrecht University.

[mbo370160-bib-0034] Gibson, M. K. , K. J. Forsberg , and G. Dantas . 2015. “Improved Annotation of Antibiotic Resistance Determinants Reveals Microbial Resistomes Cluster by Ecology.” ISME Journal 9, no. 1: 207–216. 25003965 10.1038/ismej.2014.106 PMC4274418

[mbo370160-bib-0035] Guidance, W. 2021. Ethics and Governance of Artificial Intelligence for Health. World Health Organization.

[mbo370160-bib-0036] Gupta, A. , A. S. Malwe , G. N. Srivastava , P. Thoudam , K. Hibare , and V. K. Sharma . 2022. “MP4: A Machine Learning Based Classification Tool for Prediction and Functional Annotation of Pathogenic Proteins From Metagenomic and Genomic Datasets.” BMC Bioinformatics 23, no. 1: 507. 36443666 10.1186/s12859-022-05061-7 PMC9703692

[mbo370160-bib-0037] Gupta, S. K. , B. R. Padmanabhan , S. M. Diene , et al. 2014. “ARG‐ANNOT, a New Bioinformatic Tool to Discover Antibiotic Resistance Genes in Bacterial Genomes.” Antimicrobial Agents and Chemotherapy 58, no. 1: 212–220. 24145532 10.1128/AAC.01310-13 PMC3910750

[mbo370160-bib-0038] Hamid, M. N. 2019. Transfer Learning Towards Combating Antibiotic Resistance. Iowa State University.

[mbo370160-bib-0039] Imran, L. , S. T. Rehan , and K. Y. Lee . 2022. “How Universal Health Coverage Can Curb the Escalating Antimicrobial Resistance in Pakistan: A Call to Action for the Country's Healthcare System.” Tropical Medicine and Health 50, no. 1: 86. 36376962 10.1186/s41182-022-00478-5 PMC9663288

[mbo370160-bib-0040] Jia, B. , A. R. Raphenya , B. Alcock , et al. 2017. “CARD 2017: Expansion and Model‐Centric Curation of the Comprehensive Antibiotic Resistance Database.” Nucleic Acids Research 45, no. D1: D566–D573. 10.1093/nar/gkw1004. 27789705 PMC5210516

[mbo370160-bib-0042] Kumari, S. , S. Singh , and R. S. Dhariyal . 2025. “Advancements in Diagnostic Techniques for Tuberculosis and Multidrug‐Resistant Tuberculosis (MDR‐TB): Challenges and Future Perspective of Molecular Diagnostic Methods.” Indian Journal of Preventive & Social Medicine 56, no. 1: 146–155.

[mbo370160-bib-0043] Lakin, S. M. , C. Dean , N. R. Noyes , et al. 2017. “Megares: An Antimicrobial Resistance Database for High Throughput Sequencing.” Nucleic Acids Research 45, no. D1: D574–D580. 27899569 10.1093/nar/gkw1009 PMC5210519

[mbo370160-bib-0044] Lakin, S. M. , A. Kuhnle , B. Alipanahi , et al. 2019. “Hierarchical Hidden Markov Models Enable Accurate and Diverse Detection of Antimicrobial Resistance Sequences.” Communications biology 2, no. 1: 294. 31396574 10.1038/s42003-019-0545-9 PMC6684577

[mbo370160-bib-0045] Lau, H. J. , C. H. Lim , S. C. Foo , and H. S. Tan . 2021. “The Role of Artificial Intelligence in the Battle Against Antimicrobial‐Resistant Bacteria.” Current Genetics 67, no. 3: 421–429. 33585980 10.1007/s00294-021-01156-5

[mbo370160-bib-0047] Li, Y. , Z. Xu , W. Han , et al. 2021. “HMD‐ARG: Hierarchical Multi‐Task Deep Learning for Annotating Antibiotic Resistance Genes.” Microbiome 9: 40. 33557954 10.1186/s40168-021-01002-3 PMC7871585

[mbo370160-bib-0048] Liu, B. , and M. Pop . 2009. “ARDB—Antibiotic Resistance Genes Database.” Supplement, Nucleic Acids Research 37, no. suppl_1: D443–D447. 18832362 10.1093/nar/gkn656 PMC2686595

[mbo370160-bib-0049] Lv, J. , S. Deng , and L. Zhang . 2021. “A Review of Artificial Intelligence Applications for Antimicrobial Resistance.” Biosafety and Health 3, no. 01: 22–31.

[mbo370160-bib-0051] Melo, M. C. R. , J. R. M. A. Maasch , and C. de la Fuente‐Nunez . 2021. “Accelerating Antibiotic Discovery Through Artificial Intelligence.” Communications biology 4, no. 1: 1050. 34504303 10.1038/s42003-021-02586-0 PMC8429579

[mbo370160-bib-0052] Van Messem, A. 2020. “Support Vector Machines: A Robust Prediction Method With Applications in Bioinformatics.” In Handbook of Statistics 43, 391–466. Elsevier.

[mbo370160-bib-0053] Moonesar, I. A. , and R. Dass . 2021. “Artificial Intelligence in Health Policy–A Global Perspective.” Global Journal of Computer Science and Technology 21, no. H1: 1–7.

[mbo370160-bib-0054] Nava Lara, R. A. , L. Aguilera‐Mendoza , C. A. Brizuela , A. Peña , and G. Del Rio . 2019. “Heterologous Machine Learning for the Identification of Antimicrobial Activity in Human‐Targeted Drugs.” Molecules 24, no. 7: 1258. 30935109 10.3390/molecules24071258 PMC6479866

[mbo370160-bib-0055] O'Neill, J. 2016. Tackling Drug‐Resistant Infections Globally: Final Report and Recommendations.

[mbo370160-bib-0056] Onifade, I. A. , O. A. Opasola , and A. Muazu . 2025. “Evaluation of Multi‐Drug Resistant Bacteria and High Risk of Contamination in Ready to Eat Fruits in Kano Metropolis.” Asian Journal of Medicine and Health 23, no. 4: 54–64.

[mbo370160-bib-0057] Palanikumar, P. , B. Nathan , K. Muthusamy , S. M , S. Natesan , and V. Sampathrajan . 2025. “Unravelling the Antibiotic Resistance: Molecular Insights and Combating Therapies.” Applied Biochemistry and Biotechnology 197: 3549–3580. 39964597 10.1007/s12010-025-05182-8

[mbo370160-bib-0058] Pandey, D. , B. Kumari , N. Singhal , and M. Kumar . 2020. “Baceffluxpred: A Two‐Tier System to Predict and Categorize Bacterial Efflux Mediated Antibiotic Resistance Proteins.” Scientific Reports 10, no. 1: 9287. 32518231 10.1038/s41598-020-65981-3 PMC7283322

[mbo370160-bib-0059] Rabaan, A. A. , S. Alhumaid , A. A. Mutair , et al. 2022. “Application of Artificial Intelligence in Combating High Antimicrobial Resistance Rates.” Antibiotics (USSR) 11, no. 6: 784. 10.3390/antibiotics11060784 PMC9220767 35740190

[mbo370160-bib-0060] Raisch, S. , and S. Krakowski . 2021. “Artificial Intelligence and Management: The Automation–Augmentation Paradox.” Academy of Management Review 46, no. 1: 192–210.

[mbo370160-bib-0061] Reddy, S. , S. Allan , S. Coghlan , and P. Cooper . 2020. “A Governance Model for the Application of AI in Health Care.” Journal of the American Medical Informatics Association 27, no. 3: 491–497. 31682262 10.1093/jamia/ocz192 PMC7647243

[mbo370160-bib-0062] Rives, A. , J. Meier , T. Sercu , et al. 2021. “Biological Structure and Function Emerge From Scaling Unsupervised Learning to 250 Million Protein Sequences.” Proceedings of the National Academy of Sciences 118, no. 15: e2016239118. 10.1073/pnas.2016239118 PMC8053943 33876751

[mbo370160-bib-0063] Santos‐Júnior, C. D. , M. D. T. Torres , Y. Duan , et al. 2024. “Discovery of Antimicrobial Peptides in the Global Microbiome With Machine Learning.” Cell 187, no. 14: 3761–3778.e16. 10.1016/j.cell.2024.05.013. 38843834 PMC11666328

[mbo370160-bib-0064] Seele, P. P. 2024. “Structure‐Function Relationships in the Modification of Liposomes for Targeted Drug Delivery in Infectious Diseases.” In Liposomes as Pharmaceutical and Nutraceutical Delivery Systems. IntechOpen.

[mbo370160-bib-0065] Song, B. , M. Zhou , and J. Zhu . 2023. “Necessity and Importance of Developing AI in Anesthesia From the Perspective of Clinical Safety and Information Security.” Medical Science Monitor 29: e938835–938831. 36810475 10.12659/MSM.938835 PMC9969716

[mbo370160-bib-0066] Song, L. , D. Gildea , Y. Zhang , Z. Wang , and J. Su . 2019. “Semantic Neural Machine Translation Using AMR.” Transactions of the Association for Computational Linguistics 7: 19–31.

[mbo370160-bib-0067] Song, S. , and S. Y. Shim . 2025. “Advances in Small Molecule Inhibitors Targeting the Bacterial Lipoprotein Transport System (Lol) in Gram‐Negative Bacteria.” Chemistry – An Asian Journal 20, no. 17: e00350. 10.1002/asia.202500350. 40536045 PMC12447863

[mbo370160-bib-0068] Srivastava, A. , R. Kumar , and M. Kumar . 2018. “BlaPred: Predicting and Classifying β‐Lactamase Using a 3‐Tier Prediction System via Chou's General PseAAC.” Journal of Theoretical Biology 457: 29–36. 30138632 10.1016/j.jtbi.2018.08.030

[mbo370160-bib-0069] Sun, T. Q. , and R. Medaglia . 2019. “Mapping the Challenges of Artificial Intelligence in the Public Sector: Evidence From Public Healthcare.” Government Information Quarterly 36, no. 2: 368–383.

[mbo370160-bib-0070] Thakur, N. , M. R. Alam , J. Abdul‐Ghafar , and Y. Chong . 2022. “Recent Application of Artificial Intelligence in Non‐Gynecological Cancer Cytopathology: A Systematic Review.” Cancers 14, no. 14: 3529. 35884593 10.3390/cancers14143529 PMC9316753

[mbo370160-bib-0071] Tran, M.‐H. , N. Q. Nguyen , and H. T. Pham . 2022. “A New Hope in the Fight Against Antimicrobial Resistance With Artificial Intelligence.” Infection and Drug Resistance 15: 2685–2688. 35652083 10.2147/IDR.S362356 PMC9150917

[mbo370160-bib-0072] Wang, Z. , S. Li , R. You , S. Zhu , X. J. Zhou , and F. Sun . 2021. “ARG‐SHINE: Improve Antibiotic Resistance Class Prediction by Integrating Sequence Homology, Functional Information and Deep Convolutional Neural Network.” NAR Genomics and Bioinformatics 3, no. 3: lqab066. 34377977 10.1093/nargab/lqab066 PMC8341004

[mbo370160-bib-0073] Wei, M. , A. Bostani , N. Jamalsi , H. Baqin , and P. Wen . 2025. “Bactericidal Activity of Gallic Acid Against Multidrug‐Resistant Mycobacterium Tuberculosis.” Archives of Microbiology 207, no. 9: 218. 40788398 10.1007/s00203-025-04422-z PMC12339633

[mbo370160-bib-0074] White, A. , and J. M. Hughes . 2019. “Critical Importance of a One Health Approach to Antimicrobial Resistance.” EcoHealth 16: 404–409. 31250160 10.1007/s10393-019-01415-5

[mbo370160-bib-0075] Wu, J. , J. Ouyang , H. Qin , et al. 2023. “PLM‐ARG: Antibiotic Resistance Gene Identification Using a Pretrained Protein Language Model.” Bioinformatics 39, no. 11: btad690. 37995287 10.1093/bioinformatics/btad690 PMC10676515

[mbo370160-bib-0076] Xie, G. , and J. M. Fair . 2021. “Hidden Markov Model: A Shortest Unique Representative Approach to Detect the Protein Toxins, Virulence Factors and Antibiotic Resistance Genes.” BMC Research Notes 14: 122. 33785071 10.1186/s13104-021-05531-w PMC8011099

[mbo370160-bib-0077] Zafar, A. , R. Hasan , S. Q. Nizami , et al. 2009. “Frequency of Isolation of Various Subtypes and Antimicrobial Resistance of Shigella From Urban Slums of Karachi, Pakistan.” International Journal of Infectious Diseases 13, no. 6: 668–672. 19135399 10.1016/j.ijid.2008.10.005

[mbo370160-bib-0078] Zankari, E. , H. Hasman , S. Cosentino , et al. 2012. “Identification of Acquired Antimicrobial Resistance Genes.” Journal of Antimicrobial Chemotherapy 67, no. 11: 2640–2644. 22782487 10.1093/jac/dks261 PMC3468078

